# The Effect of the Online and Offline Blended Teaching Mode on English as a Foreign Language Learners’ Listening Performance in a Chinese Context

**DOI:** 10.3389/fpsyg.2021.742742

**Published:** 2021-11-16

**Authors:** Yuhong Jiang, Yingying Chen, Jiasheng Lu, Yiqing Wang

**Affiliations:** Foreign Languages College, Shanghai Normal University, Shanghai, China

**Keywords:** online and offline blended mode, EFL junior high school learners, English listening teaching and learning activity, Quizlet task, listening performance

## Abstract

With the rapid development of digitalisation, multimedia and network-based communication technology, all walks of life are undergoing change and development driven by the application of the internet to conventional industries. Especially because of the outbreak of coronavirus disease of 2019 (COVID-19), English teaching and learning modes are undergoing revolutionary changes worldwide (Wong et al., 2020). Online courses and materials have become the norm for students, and combined with offline English learning activities, an online and offline blended learning mode has ultimately emerged (Graham, 2006; Whittaker, 2013). Whereas blended learning has been considered in several contexts, it has been less investigated in the field of blended English learning mode for the listening comprehension ability and emotional experiences of young learners of Chinese English as a foreign language (EFL) while doing the tasks. Thus, this study focussed on the achievement and experience of Chinese EFL junior high school students during blended learning using Quizlet software as the online learning tool. This study aimed to explore the following: (1) the effect of the online and offline blended mode on the learning outcomes of students and (2) the experience of students while engaging with blended tasks in terms of their learning interest, attitude, and strategy use in English listening learning. A 4-month teaching intervention involving the online and offline blended mode was conducted in English listening classes. Adopting mixed-methods qualitative and quantitative research, this study examined the engagement process of two classes of students and analysed data from their English listening tests and follow-up in-depth interviews. The results suggested that the blended activity was conducive to enhancing the listening performance of students. Moreover, the attitudes of students toward English listening learning shifted from a relatively negative engagement to a more positive one. Meanwhile, the interest of students grew and their learning strategies became more diversified. These findings have implications for English teaching and learning activity design for young learners.

## Introduction

In the current era of digital life, almost all walks of life depend on the internet (Vella-Brodrick and Klein, 2010; Amichai-Hamburger and Hayat, 2011; Choshin and Ghaffari, 2017). “We reject kings, presidents and voting. We believe in: rough consensus and running code” (Coleman, 2013, p. 171). Besides, it is observed that English learning apps on mobile devices are increasingly employed in English classes, which make students learn English more enjoyably and actively, facilitating the engagement in learning of students (Yen et al., 2018; Anthony et al., 2019; Wong et al., 2020). As Castle and McGuire (2010, p. 36) pointed out, “e-learning can enhance the quality of learning experiences because it provides flexible access to content and instruction at any time, from any place, and enables students to maintain the learning outcome equivalent to face-to-face instruction.” Digital learning games, software, and platforms have become welcome online activities that have won favour from teachers and learners. Yet, less attention has been paid to the experiences of young students during their engagement with the integration of offline and online tasks. In the context of the coronavirus disease of 2019 (COVID-19) pandemic, nationwide and worldwide school closures made online courses the norm for students, and various learning resources and types of information were delivered through the internet. Online education has become an irresistible global trend. Therefore, the research on the experience of young learners while doing online and offline blended tasks may help enrich the empirical data in this field and deepen our understanding of the benefits and challenges brought about by the blended mode, along with the difficulties of students, their emotional change, and ability shift in the process.

## Literature Review

### Defining Online and Offline Blended Teaching and Learning

Blended learning was first introduced in 1969 as a basic component of the learning system of principal distance teaching instruction of the world. The term “blended learning” emerged in the early 21st century, and it was first used to refer to “a course designed to allow workers to both continue in the workplace and study” (Sharma, 2010, p. 456). Graham et al. (2005) documented the three following definitions of blended learning: combining instructional modalities or delivery media, combining a variety of instructional methods, and combining online and face-to-face instruction. The problem with the first two definitions is that blended learning is defined so broadly that it encompasses virtually all learning systems. The third definition is characterised by the integration of instruction from two historically separate models of learning, namely, traditional face-to-face learning and distributed online learning, emphasising the central role of computer-based technology. The first appearance of the term in English language teaching (ELT) referred to any integration of face-to-face teaching with internet technology (online and offline activities/materials; Garrison and Kanuka, 2004; Whittaker, 2013). In the digital learning era, online and offline blended learning modes are defined as certain learning modes that combine contact teaching with teachers and self-contained preparation with online resources (Hubackova and Semradova, 2016). Thus, the current study takes this as the working definition of online and offline blended English learning because it reveals the nature of the blended mode—that is, blended learning can enhance the quality of learning experiences, enable students to learn English more enjoyably and actively, and facilitate the engagement of students in English learning (Castle and McGuire, 2010; Yen et al., 2018; Anthony et al., 2019; Wong et al., 2020). Learners are encouraged to complete online learning tasks and digital game-based activities, during which process they can experience the joy of dealing with digital challenges, knowledge acquisition, and enhancement of learning outcomes.

### Comparing the Blended, Online, and Offline Modes

We may suppose that there are three modes in English teaching and learning at present, namely, the conventional offline mode, the online-only mode, and the blended online and offline teaching and learning mode. Larson and Sung (2009) demonstrated that there were no significant differences among these three modes. However, the blended and online modes rated particularly highly on measures of “student satisfaction, learning effectiveness, and faculty satisfaction.” Yen et al. (2018) conducted a three-way comparison of face-to-face, online, and blended modes in an undergraduate child development course. They found that online classes could be as effective as face-to-face classes in producing satisfactory outcomes. Nevertheless, the blended mode had greater potential for improving the academic performance of students by integrating the merits of both face-to-face and online teaching. Likewise, a recent study by Yu et al. (2021) proved that both blended and offline learning were effective educational approaches to improving the critical thinking ability of students, whereas the use of blended case-centred learning showed promising results in improving the academic performance of students.

Overall, each mode has its own features and advantages. Nonetheless, the blended teaching mode is more effective than the other two because it capitalises on the strengths of the two and leads to positive student outcomes (Ho et al., 2016).

### Studies on Blended Teaching and Learning Modes in the English as a Foreign Language Context

Previous studies displayed two major themes, namely, experience of learners on learner autonomy and motivation (Garrison and Kanuka, 2004; Ige and Hlalele, 2017; Anthony et al., 2019; Dolores et al., 2019), and the impact on the language ability, including reading, writing, listening, and vocabulary of learners (Caruso et al., 2017; Kazakoff et al., 2018). Regarding the first theme, studies focus on the experience of learners, examining how blended learning has the potential to enhance both the effectiveness and efficiency of meaningful learning experiences (Garrison and Kanuka, 2004, p. 95). In this line of research, Wong et al. (2020) demonstrated that in contrast with the traditional learning, the blended mode had positive effects on both learner autonomy and motivation in secondary school English classes. Moreover, the research findings of Anthony et al. (2019) revealed that the impact of blended learning on the effectiveness of learners was positively predicted by achievement, engagement, involvement, retention, and cognitive outcome. In another study, Dolores et al. (2019) explored how to transform traditional face-to-face learning into blended learning and ultimately develop the initiative in engagement of students through both in-class and online approaches, which was also time effective for teachers. Furthermore, Ige and Hlalele (2017) conducted a blended learning experiment in junior high schools in the Ondo region of Nigeria and found that blended learning could help create a student-centred lesson and that the learning effect was greatly improved. Bernard et al. (2014) examined the effect of blended learning in higher education through a meta-analysis of comparative studies of blended learning and classroom instruction.

Another line of research involves a more specific study of the impact on the language ability of students. Kazakoff et al. (2018) confirmed that the blended learning approach contributed to the development of reading ability in learners of English. Likewise, Caruso et al. (2017) discussed the integration and effect of blended learning on the development and assessment of listening skills in a second language. In addition, Miyazoe and Anderson (2010) delved into the effect of blended courses on learning outcomes and perceptions of students to online writing with the employment of three different online writing tools in an English as a foreign language (EFL) blended learning setting, and the results suggested a positive effect on the language learning progress of students. In the same line of research, Rusanganwa (2013) contrasted the teaching effects of computer-assisted language teaching and offline teaching in college English for special purposes (ESP) vocabulary teaching. Accumulating evidence has shown that the blend task plays a crucial role in the internalisation of vocabulary of students.

In studies of blended learning in the Chinese EFL context, one line of research deals with the effects of blended learning tasks on learner attention, confidence, and attitude in learning English, while another focuses on the impact of blended learning on language ability (reading, writing, listening, and vocabulary) of students. Ma and Lee (2021) illustrated that blended learning outperformed mere online or offline learning in enhancing attention, confidence, and satisfaction perceptions of students. Wang (2021) also indicated that blended learning had an overall positive impact on the English conversation performance of students, and that students had a positive attitude toward the blended course. Yao (2019) found that a blended learning environment contributed to improving English acquisition of Chinese adult learners, especially their English writing abilities. Likewise, Zhou (2018) confirmed that the blended learning mode could significantly improve the English writing ability of students. Cui (2014) investigated the impact of a blended approach aimed at improving the listening and speaking skills of students. The results proved that it was effective not only in helping improve listening and speaking skills but also in enhancing learner autonomy. Jia et al. (2012) elucidated that an English blended learning class with the individualised vocabulary acquisition and assessment system was conducive to improving the performance of students in vocabulary acquisition and ordinary testing. Similarly, Yang (2012) illustrated that blended learning was effective in enhancing the reading proficiency of students. Wang (2011) explored the impact of blended learning on the English writing proficiency and motivation of 170 Chinese non-English major college students. The results proved that blended learning had a positive impact on the improvement of English writing proficiency and learning motivation, especially intrinsic motivation.

From the aforementioned studies, it is found that blended learning involves a complex fusion of online and offline learning environments, learning resources, strategies, and evaluations. It is indisputable that these elements complement each other to help students master essential knowledge and develop their language efficiency. Nevertheless, some deficiencies need to be addressed. For example, in blended learning, the workloads of teachers would increase, and teachers and students are required to master information technology, but they lack sufficient knowledge and operating experience of online software. What is more, when the online operating platform is unstable, teaching effectiveness can be reduced. Thus, this study is committed to exploring the effect brought about by integrating online and offline tasks.

There is still some space for further exploration in this field for three reasons. First, most prior studies (e.g., Graham, 2006; Garrison and Vaughan, 2008) have only dealt with some aspects of the blended mode, ranging from its definition, categories, framework, principles, and guidelines to its application in multiple subjects and diversified areas. The experience of young learners immersed in blended practice in the Chinese EFL technology-based context has not yet been examined. Second, although a few studies have documented that the blended activity produces positive results on student performance, it remains underexplored how it may exert an effect on the English listening performance of young learners, how different groups of students may differ in listening achievements, and what students experience during involvement with the blended tasks in terms of learning attitude, interest, and strategy use. Third, the research settings in previous studies are mainly embedded in English as the mother tongue or first language context rooted in English culture, and samples mainly involve college students. They lack a probe into the specific linguistic and socio-cultural context in which English is learned as a foreign language, which restricts enriching the body of literature on blended mode research in language learning.

Given the limited studies in this area concerning the complex experiences of students (Sharpe and Benfield, 2005) or the categories of outcomes obtained with the help of the blended learning mode (Alexander, 1999; Dowling et al., 2003; Lim and Morris, 2009), it is necessary to employ a mixed-methods approach to further investigate the impact of blended tasks on English listening comprehension of junior high school students and focus on the experience of students in terms of any possible shift in learning interest, attitude, and strategy use while completing listening tasks. The study is worthwhile because of the current educational needs in the global epidemic situation. This may suggest potential implications for the current design of teaching and learning during the post-pandemic era.

A constructivist perspective is adopted in this study because it “views learning as an active process in which learners construct their own understanding and knowledge of the world through action and reflection” (Williams and Burden, 1997), which coincides with the nature of the actual instructional content that is formatted for online and mobile learning. Since our study is concerned with the interpretation and description of the experiences of engagement of participants in online and offline blended practice and how the individual participants deconstruct and reconstruct their thoughts and practice in the context of a new initiative, namely, blended learning, it is interpretive and constructive in nature. Given that a classroom is a social setting where teachers and students interact with each other, this study conforms to a constructivist epistemology. The study sets out to interpret the experiences of participants in the local context of change. The constructivist perspective helps to scaffold the whole process and to incorporate and allow for a multiplicity of points of view. These considerations led us to embed this study within a constructivist research paradigm (Denzin and Lincoln, 2005, p. 22).

## Research Design

### Research Questions

To cope with the mentioned inadequacies in prior studies, the present research aims to address the following research questions:

(1) How do online and offline blended modes affect the performance of students in English listening comprehension?
(2) What do the students experience while engaging with the online and offline blended task in terms of learning interest, attitude and strategy use in English listening learning?

### Research Participants

The study uses the cluster and convenience sampling methods, which allow us to select participants for our purpose (Denscombe, 2007) and to build up a sample that will satisfy our specific needs (Cohen et al., 2005; Jiang, 2012, 2017). The participants were students in Grade 9 of a junior high school in Shanghai, China, with 42 students from Class One and 53 from Class Two. Class One was selected as the experimental class (EC) and Class Two as the control class (CC). All the students began to learn English from Grade 1. The teachers of the two classes voluntarily participated in the study. The teacher in the EC received training before the intervention to learn to conduct English listening teaching activities via the online and offline blended mode, while the other teacher continued to use a traditional face-to-face offline mode in the CC. Grade 9 students were targeted because they had mastered a certain amount of English listening skills compared with Grade 8 and Grade 7 students. In addition, they could feel freer to accomplish somewhat complicated listening tasks in online learning. To make the intervention statistically meaningful, it was necessary to analyse the level of English listening of participants before conducting the treatment. As shown in Table 1, the pre-test scores of listening proficiency between the EC and the CC were not significantly different [*t*(73) = 0.058, *p* > 0.05]. Therefore, the English listening comprehension levels of the two classes of samples were considered similar prior to the teaching treatment.

**TABLE 1:** Independent-samples *t*-test: Comparison of the pre-test scores of students in listening comprehension between the EC and the CC.

**Pre-test Scores**	**Experimental**		**Controlled**		**MD**	***t*(73)**	**Sig.**
	**(*n* = 42)**		**Class (*n* = 53)**				
	***M***	**SD**	***M***	**SD**			
	16.50	5.13	16.45	4.52	0.05	0.058	0.954
							

This study conducted semi-structured interviews to triangulate the quantitative results and to obtain a full and deeper understanding of the perceptions and feelings of the participants for online and offline blended tasks in English listening. Ten interviewees were selected from the EC, and the top five scorers and the bottom five scorers on the post-test contributed their feedback. The interviewees were selected for our purpose according to the criteria of purposive sampling (Denscombe, 2007). Table 2 shows the detailed information of the interviewees. For ethical reasons, pseudonyms were used to refer to them. The researchers made a commitment to keep the information related to their personal privacy secret. The researchers notified the participants of the research aims and procedures of the blended learning tasks, asked them to sign the letter of consent, and told them that they could choose to withdraw from the learning task at their discretion.

**TABLE 2:** Detailed information of the interviewees.

**Name**	**Gender**	**Age**	**Academic degree**	**Code**
Eric	Male	15	Grade 9	IN1
Helen	Female	15	Grade 9	IN2
Iris	Female	15	Grade 9	IN3
Johnson	Male	14	Grade 9	IN4
Rebecca	Female	15	Grade 9	IN5
Cindy	Female	15	Grade 9	IN6
Anna	Female	15	Grade 9	IN7
Danny	Male	15	Grade 9	IN8
Jerry	Male	15	Grade 9	IN9
Andy	Male	15	Grade 9	IN10

### Research Method

To address the inadequacies mentioned above, it was necessary to adopt a mixed-methods approach to obtain quantitative data in the form of independent-samples *t*-tests. The quantitative data were probed in greater depth through analysis of a range of qualitative data in terms of the interview transcripts, extending the sampling to junior high school students, and focussing on the differences in the effects that the traditional and blended learning modes might bring to the learning outcomes and learning emotions of different groups of students.

The basic rationale for using the mixed-methods approach in the current study was that by combining qualitative and quantitative methods, one could utilise their respective strengths and escape their respective weaknesses (Tashakkori and Teddlie, 1998), so this approach had been widely used in both basic and applied research (Lund, 2012). Particularly for this study, there were two reasons for choosing this approach. First, as Onwuegbuzie and Leech (2005, p. 375) claimed, “Monomethod research is the biggest threat to the advancement of the social sciences.” They viewed mixed-methods studies as superior to investigations produced by either quantitative or qualitative research alone. Likewise, Mertens (2005) argued that mixed methods “have particular value when we want to examine an issue that is embedded in a complex educational or social context” because they help broaden the scope of the investigation and enrich the ability of researchers to draw conclusions about the problem under study. Second, the obvious benefit of a mixed-methods approach was that “seeing things from a different perspective and the opportunity to corroborate findings can enhance the validity of data” (Denscombe, 1998, p. 85). To corroborate findings meant verifying and validating one set of findings against another (Sandelowski, 2003), namely, triangulation. This approach can allow access to a fuller understanding of the multifaceted nature of the target phenomenon in this study.

### Research Instruments

This study used the three following instruments to collect data that would reflect perception, practice, and achievements of students in doing blended tasks: pre and post-tests, follow-up semi-structured interviews, and an online English learning tool, namely, the Quizlet website platform.

#### Test (Shanghai Senior High School Entrance Examination)

The listening parts of the *Shanghai Senior High School Entrance Examination* from 2018 and 2019 were selected as the pre and post-tests in the research, respectively, and they were utilised as tools to collect quantitative data to evaluate the listening performance of students. We chose the *Shanghai Senior High School Entrance Examination* for the two following reasons: first, this examination paper always enjoys high authority, credibility, and reliability because the teaching and research experts of Shanghai basic education are responsible for designing the tests every year; second, the examination paper compilers accumulate practical experiences and feedback from teachers and students for improving it annually, and it has been proved to respond to the practical needs of Grade 9 students and fit their true English levels.

The structures of the listening tests were divided into four parts, from the simpler ones to the more difficult ones, including the following: *Part A: Listen and choose the right picture*; *Part B: Listen to the dialogue and choose the best answer to the question*; *Part C: Listen to the passage and tell whether the following statements are true or false*; and *Part D: Listen to the dialogue and complete the following sentences*. The scoring criteria, set up by the Shanghai Municipal Education Commission, are listed in Supplementary Appendix 1.

#### Semi-Structured Interview Protocol

A semi-structured interview protocol was designed to obtain qualitative data to probe into the experiences and emotions of participants while completing the listening tasks. To ensure a better understanding of the items, the interviews were conducted in the mother tongue of the participants, Chinese. The interviews were recorded and subsequently transcribed. Then, the participants were asked to double check if there was any error in the transcripts based on their retrospective self-reports, and the authors modified the wording accordingly. Each interview lasted about 40 min.

#### Quizlet Learning Tool

The Quizlet learning tool is a popular learning software and website platform for motivating learners to learn English through interesting games. This study chose it as the instrument for doing online listening learning tasks because of its popularity among young people. On the one hand, users can do a variety of interesting games to learn language to solve learning problems by taking advantage of the Quizlet website to memorise vocabulary in the word cards and use models to test the knowledge repertoire of users. On the other hand, teachers cab share micro-lectures on the online learning community, and students can log in to the Quizlet website to watch the micro-lectures and complete listening quizzes. The test scores and study time of students can be recorded using the tool system.

### Research Procedures

#### Intervention Training

The teacher in the targeted EC class carried out blended online and offline English listening teaching during the intervention. She participated in the blended teaching mode promoting training, which took place in the summer preceding the intervention, with the purpose of helping the teacher to become more blended teaching oriented to enact blended instructional behaviours and less solely face-to-face teaching in the classrooms. A 4 h workshop was conducted by the authors. This workshop included an overview of blended mode–supportive teaching, provided empirical evidence from previous studies on its benefits compared to face-to-face teaching, teaching cases, and examples of blended teaching behaviours, and requested the teacher to reflect on the two kinds of teaching behaviours in her English listening instruction.

#### Intervention and Data Collection

It took 4 months for this study to perform the intervention in the academic year 2019–2020. Throughout this period, the teacher in the treatment class applied online and offline blended modes in English listening teaching.

The data collection period lasted for 4 months during the intervention, from September 2019 to January 2000, and it was completed in several stages. At the beginning, the pre-test examining English listening proficiency of students was conducted in the two classes, EC and CC, while the post-test was completed after the 4-month intervention. Students were required to finish the tests within 30 min. The tests were scored, and different results between the EC and CC were analysed by employing SPSS 26.0.

*Oxford English, Shanghai Edition* was chosen as the teaching material in this research because it is the mandated textbook used in most junior high schools in Shanghai. When selecting the appropriate blended task, the researchers sought advice from the teachers and students regarding the fun and informativeness of the material. Based on their suggestions and feedback, *The Adventure of Tom Sawyer* in Module 4, Unit 7, Year 9 was finally selected as the sample for the teaching case of the intervention. On the one hand, the wonderful plot of the story was expected to stimulate interest of students in listening learning. On the other, the content analysis of the adventure would cultivate good characters in students, such as integrity and courage, and make them ready to help others.

The students were required to complete Quizlet learning tasks within class hours during the blended listening teaching intervention. After students had finished the tasks, their performance was recorded and assessed by the Quizlet system. The teacher would check the online work and achievement of students and give feedback later.

The follow-up interviews were conducted after implementing the intervention to elicit the feelings and perceptions of students regarding their personal listening learning experience while engaged in the blended mode. A total of 10 students, representing the top five and bottom five scorers in the EC, were invited to participate in the interviews.

### Data Analysis

SPSS 26.0 was employed to conduct the data analysis. To address Research Question (RQ) 1 on the impact of the blended learning tasks on improving the performance of young learners in English listening comprehension, independent sample *t*-tests and paired samples tests were undertaken using SPSS 26.0. The study mainly focussed on contrasting the results of pre-test and post-test listening performances between the EC and the CC and within the EC. The scores of the listening parts of the tests were analysed, and the results of contrasting the differences between the EC and the CC were gained.

To probe the prominent features of the experience of students in doing blended learning tasks and differentiate the factors that contribute to the change of achievement, the correlations between different parts of the pre-tests, and post-tests needed to be determined. Pearson correlation analysis was performed in this study to identify the variables of different parts of the pre-tests and post-tests in the EC and CC.

In addressing RQ 2 on investigating the experience of students while engaging with the blended listening tasks in terms of learning interest, attitude, and strategy use, the study used thematic analysis. Codes were created based on the RQs and on the themes and categories that emerged from the data. The coding framework of thematic analysis and a “grounded approach” (Glaser and Strauss, 1967; Strauss and Corbin, 1998) were used to categorise the data with the themes that emerged from the responses of interviewees. Qualitative content analysis (Miles and Huberman, 1994) was used as the data analysis strategy, aiming to disclose how individual students understood or interpreted the issues related to the change of interest and attitude and what factors were involved in this change. The authors employed the following analytical strategies: transcribing the interview recounts, noting emerging patterns and themes, clustering and counting the patterns, conducting contrasts, and comparisons to look for differences between the two different groups of students, noting relations between variables and building a logical chain of evidence (Jiang, 2012). Finally, the interviewees checked the excerpts and offered revisions where there was a conflict with their thoughts.

In short, the qualitative thematic evidence informed the discussion of the evolution of ideas and behaviours on a cross-case basis. Both the quantitative and qualitative data contributed to a full interpretation of the experiences of the students.

## Implementation of the Online and Offline Blended Mode in English Listening Teaching

### Teaching Procedures of the Experimental Class

Throughout the period of intervention, the teacher in the treatment class applied the blended teaching mode in English listening, considering the principles and evaluation of blended learning based on Vaughan et al. (2017). The teaching procedures of the blended mode and the traditional one in English listening teaching are explained in Supplementary Appendix 2, 3, respectively. *The Adventure of Tom Sawyer* in Module 4, Unit 7, Year 9 of *Oxford English, Shanghai Edition* was selected as the sample for both teaching cases.

In the online teaching procedure, first, the teacher recorded a micro-lecture of 5–10 min in length that was centred on the teaching objectives, focus and difficulties, text analysis, and use of vocabulary. Then, the teacher shared it on the online learning community, the Quizlet website. The students were required to log into the Quizlet website to finish the tasks and share their difficulties, study plans, questions, and opinions in the online discussion community. The test scores, study time on the website, study plan, and opinions of the students were shown in the discussion community on the website. Finally, the teacher provided feedback on the listening outcomes of students according to the recording of the tool system.

It is worth noting that the online system recorded the time that students spent on each word and then automatically formed questions related to these words and explanations. Therefore, on the one hand, the listening learning was personalised by students using strategies that were fit for them with internet technology, which would help improve their performance through completing personalised online listening tasks. On the other, the teacher could conduct a systematic investigation of the actual listening level of students before implementing the offline teaching, allowing the teacher to explore the potential development of their listening ability.

In the design of offline teaching procedures (during class) within the whole blended teaching task, there were seven steps to implement the teaching (see Supplementary Appendix 2). The teacher helped students consolidate and internalise listening skills and strategies through various tasks and activities. The tasks were designed based on the listening learning outcomes of students assessed by the Quizlet tool. In the whole procedure, the teacher always paid attention to the reactions of students and gave timely and personalised guidance.

In the design of online revision tasks (after class), students were required to log into the Courseware App to complete the assigned tasks based on the recordings and finish the listening exercises from the easier ones to the more difficult ones. Then, by analysing the scores they obtained, the online system designed several related listening materials to match the performance of each student. What was more, a short discourse with a topic relevant to the listening materials was displayed on the online system, and students were asked to provide their answers on the topic within 3 min and then upload them to the Quizlet system. Finally, students needed to listen to the daily news on the app and then make news reports in groups in the next class.

### Teaching Procedures of the Controlled Class

The traditional offline face-to-face mode was implemented in the CC with 53 students. The teaching followed the three teaching steps of a pre, while, and post (PWP) approach. A teaching case indicating the teaching procedure is presented in Supplementary Appendix 3.

## Results

In response to RQ 1, independent samples *t*-tests, paired samples tests, and correlations analysis were deployed to analyse the data of the pre-test and post-test to compare each part of the scores gained by the EC and CC. To address RQ 2, the interview data were used to discuss the personal perceptions, cognition and experiences of students in terms of learning interest, attitude, and strategy use in coping with English listening tasks during their engagement with the blended learning mode.

### Results of RQ 1: The Impact of Online and Offline Blended Tasks on the English Listening Comprehension Ability of Students

Independent samples *t*-tests, paired samples tests, and correlation analysis were used to investigate the different impacts of blended tasks on the English listening comprehension of students between the EC and the CC.

#### Results of the Independent Samples *t*-Tests

Table 1 demonstrates that there was no significant difference in English listening proficiency between the EC and the CC before the intervention [*t*(73) = 0.058, *p* > 0.05]. Inspections of the two group means indicated that the average pre-test score in the EC was only 0.05 points higher than the score in the CC. Therefore, the levels of English listening of the participant students in the EC and CC were nearly the same before the treatment.

Table 3 indicates that there was a significant difference between the EC and the CC in listening performance after conducting the intervention [*t*(93) = 7.069, *p* < 0.05]. Inspections of the two group means indicated that the average post-test score in the EC was significantly higher than that in the CC (MD = 3.96). This means that the listening ability of students in the EC had improved. In addition, compared with the pre-test scores in Table 1, the mean of the post-test in the EC had increased by 6.24 points and the mean of the post-test in the CC had only increased by 2.32 points. Thus, the rate of increase in the EC was much higher than that in the CC.

**TABLE 3:** Independent-samples *t*-test: Comparison of students’ post-test scores of listening comprehension between the EC and the CC.

**Post-test Scores**	**Experimental**		**Controlled**		**MD**	***t*(93)**	**Sig.**
	**Class (*n* = 42)**		**Class (*n* = 53)**				
	***M***	**SD**	***M***	**SD**			
	22.74	2.61	18.77	2.79	3.96	7.069^*^	0.000

*p < 0.05.

#### Results of the Paired Samples Tests

As mentioned above, the structure of the listening tests consisted of four parts (A, B, C, and D). To verify whether the employment of blended tasks improves the English listening outcome of students in the four types of test questions, paired samples tests were used to analyse the differences in the scores of the four components between the EC and the CC. In the tables, A1, B1, C1, and D1 refer to Parts A, B, C, and D in the pre-test, whereas A2, B2, C2, and D2 refer to Parts A, B, C, and D in the post-test.

Tables 4–7 indicate that there were significant differences between the pre-test and post-test on the scores of listening of students in the EC. Parts A, B, and C in the EC had clearly improved after the intervention [*t*(41) = −7.646, *p* < 0.05; *t*(41) = −10.032, *p* < 0.05; *t*(41) = −8.713, *p* < 0.05]. The most salient change was found in Part D [*t*(41) = −15.091, *p* < 0.05], which means that the listening outcome of students was greatly enhanced in Part D compared with the other parts of the post-test. Inspections of the two group means indicated that the average scores of Parts A, B, C, and D of the English listening of students before the intervention (4.24, 4.67, 4.26, and 3.33, respectively) were significantly lower than those of the post-tests (5.64, 6.17, 5.38, and 5.55, respectively). The differences between the means were −1.41, −1.50, −1.12, and −2.21 points on the 50-point test. The results indicated that the blended teaching mode had a positive impact on English listening skills, levels, and achievement of young learners.

**TABLE 4:** Paired samples test: Comparison of pre-test and post-test scores of Part A of listening comprehension in the EC.

**Test Scores**	**A1**		**A2**		**MD**	***t*(41)**	**Sig.**
	***M***	**SD**	***M***	**SD**			
	4.24	1.41	5.64	0.76	−1.41	−7.646^*^	0.000

*p < 0.05.

**TABLE 5:** Paired samples test: Comparison of pre-test and post-test scores of Part B of listening comprehension in the EC.

**Test Scores**	**B1**		**B2**		**MD**	***t*(41)**	**Sig.**
	***M***	**SD**	***M***	**SD**			
	4.67	1.22	6.17	0.85	−1.50	−10.032^*^	0.000

*p < 0.05.

**TABLE 6:** Paired samples test: Comparison of pre-test and post-test scores of Part C of listening comprehension in the EC.

**Test Scores**	**C1**		**C2**		**MD**	***t*(41)**	**Sig.**
	***M***	**SD**	***M***	**SD**			
	4.26	1.17	5.38	0.62	−1.12	−8.713^*^	0.000

*p < 0.05.

**TABLE 7:** Paired samples test: Comparison of pre-test and post-test scores of Part D of listening comprehension in the EC.

**Test Scores**	**D1**		**D2**		**MD**	***t*(41)**	**Sig.**
	***M***	**SD**	***M***	**SD**			
	3.33	1.63	5.55	1.21	−2.21	−15.091^*^	0.000

*p < 0.05.

As shown in Tables 8–11, the students in the CC scored higher on Parts B, C, and D on the post-test than on the pre-test [*t*(52) = −2.971, *p* = 0.004 < 0.05; *t*(52) = −4.060, *p* < 0.05; *t*(52) = −4.852, *p* < 0.05], and their performance on Part A was the highest [*t*(52) = −6.713, *p* < 0.05]. Inspections of the two group means indicated that the average scores on Parts A, B, C, and D for the English listening of students before the intervention (4.23, 4.91, 3.85, and 3.47, respectively) were moderately lower than those of the post-tests (5.09, 5.28, 4.32, and 4.08, respectively). The differences between the means were −0.87, −0.38, −0.47, and −0.60 points on the 50-point test. However, the degrees of difference were not as great in the CC compared with the EC. In this sense, compared with the traditional solely offline mode, the blended learning mode was more effective in improving the listening performance of students.

**TABLE 8:** Paired samples-test: Comparison of pre-test and post-test scores of Part A of listening comprehension in the CC.

**Test Scores**	**A1**		**A2**		**MD**	***t*(52)**	**Sig.**
	***M***	**SD**	***M***	**SD**			
	4.23	1.12	5.09	1.06	−0.87	−6.713^*^	0.000

*p < 0.05.

**TABLE 9:** Paired samples-test: Comparison of pre-test and post-test scores of Part B of listening comprehension in the CC.

**Test Scores**	**B1**		**B2**		**MD**	***t*(52)**	**Sig.**
	***M***	**SD**	***M***	**SD**			
	4.91	1.09	5.28	0.72	−0.38	−2.971^*^	0.004

*p < 0.05.

**TABLE 10:** Paired samples-test: Comparison of pre-test and post-test scores of Part C of listening comprehension in the CC.

**Test Scores**	**C1**		**C2**		**MD**	***t*(52)**	**Sig.**
	***M***	**SD**	***M***	**SD**			
	3.85	0.77	4.32	0.70	−0.47	−4.060^*^	0.000

*p < 0.05.

**TABLE 11:** Paired samples-test: Comparison of pre-test and post-test scores of Part D of listening comprehension in the CC.

**Test Scores**	**D1**		**D2**		**MD**	***t*(52)**	**Sig.**
	***M***	**SD**	***M***	**SD**			
	3.47	1.09	4.08	1.21	−0.60	−4.852^*^	0.000

*p < 0.05.

We may draw a conclusion from the above data that the intervention of the blended learning mode made the teaching effect improve a lot in the EC. This result was also confirmed by the results of the interview data of the participants.

#### Results of the Correlation Analysis

Tables 12, 13 illustrate that the values of correlation in each pair were significantly higher than 0.5, which indicates a significant correlation between the different parts of the pre-test and post-test. Thus, there existed good reliability and validity in the tests, and this verified a positive achievement in the listening task or students.

**TABLE 12:** Paired samples correlations of the four different Parts of A, B, C, and D of the pre-test and post-test in the EC.

		***N***	**Correlation**	**Sig.**
Pair 1	A1 and A2	42	0.537	0.000
Pair 2	B1 and B2	42	0.616	0.000
Pair 3	C1 and C2	42	0.730	0.000
Pair 4	D1 and D2	42	0.816	0.000
				

**TABLE 13:** Paired samples correlations of the four different Parts of A, B, C, and D of the pre-test and post-test in the CC.

		***N***	**Correlation**	**Sig.**
Pair 1	A1 and A2	53	0.629	0.000
Pair 2	B1 and B2	53	0.548	0.000
Pair 3	C1 and C2	53	0.341	0.012
Pair 4	D1 and D2	53	0.692	0.000

### The Results of RQ 2: The Three Aspects of the Experience of Students in Terms of Learning Interest, Attitude, and Strategy Use While Engaging With Online and Offline Blended English Listening Tasks

After accomplishing the intervention, the students offered their feedback about their experience in the blended online and offline learning tasks from three perspectives, namely, enhancement in their learning interest, change of their attitude, and improvement in their strategy use. These interview data supported the findings from the tests.

#### The Experience of Students Related to Learning Interest While Engaging With Online and Offline Blended English Listening Tasks

The interview data demonstrated a few differences between the two groups of students (the top five scorers and the bottom five scorers) in the post-test in the EC. For the first question in the interview protocol, “Which learning mode is more suitable for you in English listening?”, all the interviewees reported that they preferred the blended learning mode, which made the English listening learning process more interesting and flexible. Among them, six students (Interviewees 2, 3, 6, 8, 9, and 10) emphasised the advantage of and their preference for the online video clips and micro-lectures. Interviewee 3 related,

*From my point of view, to a great extent, the online and offline blended learning modes stimulated my learning interest in English listening. For example, the competition for personal learning products shared in the online discussion community motivated my passion for English listening learning. In addition, the video about wild life protection made me feel that the real purpose of the class was to call on us to protect the wild life instead of simply learning English listening skills. It was more vivid and interesting than merely looking at the pictures printed in the textbooks and playing recordings. I think I will take the initiative to study English and spend more time learning English by doing the blended tasks (IN3, Dec. 2019).*

This comment shows that the blended activities motivated Interviewee 3 to learn English, listening more passionately and actively. He was more interested in his online task because it stimulated him to go beyond the skills to explore the connotation of the texts in depth.

Echoing the previous point, three other students (Interviewees 1, 4, and 7) reported their greater interest in the online discussion community. As Interviewee 4 stated,

*I think that the discussion community provided an atmosphere where you could speak freely about your views and opinions, which you may be too nervous to express clearly and appropriately in the offline class. So, blended learning really benefited me a lot (IN4, Dec. 2019).*

In addition, Interviewee 5 claimed that blended learning tasks provided her with adequate resources to choose from, so she paid more attention to the content she was really interested in, which promoted her deep learning activity. She stated,

*I found myself digging deeper into ideas and tasks that interested me, so my learning seemed very personal (IN5, Dec. 2019).*

In short, during blended learning, all 10 students enjoyed the tasks, and this mode made their English listening learning process more interesting and diversified. In this sense, they were not significantly different in their perspectives on their change in learning interest. However, they all perceived the transformation in their interest now, compared with that in the traditional teaching context. The enhancement in the scores of the post-test results also deliver evidence for the shift of the students to taking more interest in English listening learning.

#### The Experience of the Students Regarding Learning Attitude While Engaging With Online and Offline Blended English Listening Tasks

The interviews examined the lack of conformity between the two groups of students regarding their attitudes toward the blended tasks. When asked the third question, “How do you evaluate the online offline blended learning mode? What are its advantages and disadvantages?”, five respondents (Interviewees 2, 3, 5, 6, and 10) reported that doing the blended learning tasks stimulated them to engage in the listening activities more positively, and thus, they experienced extensive benefits. For instance, Interviewee 6 explained:

*I like the blended learning mode. The implementation of the online and offline blended learning mode is different from the traditional one. I just learned what the teacher required and finished the listening tasks and I was passively involved in English listening learning before; now, I learn listening materials on the internet first and complete listening tasks by myself, which helps me get a lot of satisfaction from learning so that I have a strong desire to explore unknown fields of knowledge. I participate in English listening more actively than before. Therefore, I think that my attitude toward English listening learning also changed via employing the blended learning activity (IN 6, Dec. 2019).*

The response by Interviewee 6’s displayed his preference for the blended activity compared with the more traditional one. He clearly realised that his attitude underwent a change, from more passive learning to more autonomous and independent learning. Likewise, another two participants (Interviewees 7 and 9) claimed that the online environment offered them enough room to rehash ideas and complete the task, so that they could remain free of doing tasks. Interviewee 7 responded:

*In the online environment, you were given time to think, to form your own interpretations, to hash and rehash ideas. Thoughts were not overshadowed by someone in the class where conversations were ongoing (IN 7, Dec. 2019).*

As for the remaining interviewees (Interviewees 1, 4, and 8), they sensed that the rich and profound online resources expanded their horizons and led to the formation of their values, enabling them to learn more actively and understand themselves better than before. A comment by Interviewee 8 gives an example of this perception:

*Instead of passive learning and listening in face-to-face classes, I actively engaged in every activity and task in the online course. And I look at things very differently now—from the classroom to world events and issues*—*because of the abundant listening materials shared on the internet. I better understand individuals who are not like myself. I understand myself better as well (IN 8, Dec. 2019).*

From the responses, we may see that after the intervention, no variances were found in the perception of students of their attitude toward the blended tasks. The interviewees all felt satisfied with the achievements gained through the blended learning tasks, asserting that blended learning increased their strong desire to learn English listening skills, to consider topics from various perspectives and to actively participate in the class discussion. Like Osguthorpe and Graham’s (2003) findings, our results showed that the blended task is cost effective, which can facilitate revision of students, promoting deep learning, and better performance. Students declared that they developed more positive attitudes because of the unique features of blended learning, such as the easy accessibility and availability of course materials, the use of web-based or electronic tools for communication, and the friendly community of discussion, which all catered to the needs of students for convenient communication. Thus, their preference for this mode was undoubtable. The higher scores on the post-test also provided evidence for the shift by students to taking a more positive attitude toward studying English listening skills.

#### The Experience of Students Concerning Learning Strategy Use While Engaging With Online and Offline Blended English Listening Tasks

Differences in learning strategy use between the higher achievers and lower achievers were confirmed in the interviews. The responses of the students to the second question (“How do you solve the problems in the process of blended listening learning?”) and the fifth question [“What is the focus of your listening learning (vocabulary, reading, or material content) in the online English listening?”] further helped to understand the perception of students of any shift in their use of listening learning strategies. Seven respondents mentioned that the blended learning activity changed their learning strategies. Among these respondents, five (Interviewees 1, 4, 5, 6, and 9) responded that their approaches to memorising new words had changed because of engagement in the blended tasks, which further improved their efficiency in terms of previewing and reviewing the listening materials. Interviewee 9 made the following comments:

*My problem with English listening learning is due to a shortage of vocabulary. I can’t recognise the complicated words and fail to grasp key information from the recordings when the theme of listening materials is the news. Therefore, my English listening skills are related to the acquisition of vocabulary. After doing the online learning tasks, the way that I memorised new words transformed from solely employing textbooks to the use of apps. Every week, I used the laptop to log in to the Quizlet website to learn and memorise vocabulary in the word cards, analyse the examples of words and answer questions related to the words that had been listed beforehand. And in my spare time, I utilised my mobile phone to log into the Quizlet app to do exercises (IN 9, Dec. 2019).*

This interview demonstrated that, targeting the change of strategy use, Interviewee 9 adopted a new method of taking advantage of the Quizlet learning tool and other apps to memorise words and do exercises. Like in Zhang and Pérez-Paredes (2019) findings, our results showed that vocabulary learning and mobile dictionary applications can be rated as the favourite online resources of students because of their unique features of having a fun design, including interesting games and allowing easier access.

Interviewee 10 expressed his transformation in the use of strategy. He was better able to adopt more effective strategies to cope with difficulties and figure out problems in the process of doing blended activities than before:

*Actually, the online and offline blended tasks changed my way of solving listening learning problems. The blended mode dramatically reduced my learning pressure, and my listening strategies also changed. The teacher integrated internet technology into English listening teaching, which directed me to comprehend important and difficult points of learning. After class, unsolved listening problems could be worked out by communicating with classmates in the Quizlet discussion community and searching the answers on the learning tool. Therefore, I sensed a change in my listening-learning strategies in the process of doing blended tasks (IN 10, Nov. 2019).*

From a different perspective, Interviewee 2 attended to making full use of the multimodality in the online video clips, such as body language and tone. This new method facilitated his understanding of the meanings of the listening materials more than before the blended intervention:

*People’s facial expressions, body language, tone and intonation in the video clips helped me understand the content of the listening materials and learn the new vocabulary more vividly and easily (IN 2, Nov. 2019).*

However, the other three respondents showed differences in that they held that there was no significant change in their learning strategy. In their view, this was due to their limited English learning ability and lower language level.

In summary, there was a consensus among the respondents that their engagement in the blended learning tasks helped them become more skilful in taking advantage of learning resources, overcoming listening difficulties, and figuring out complicated problems. Knowing how to optimise learning methods and deal with learning difficulties was conducive to cultivating the ability of students to use strategies, which in turn supported their learning outcomes. Thus, the results of the interviews corroborated that the blended tasks shifted the use of listening strategies from single strategies by interviewees to more diversified strategy types.

The students confirmed their gains in this blended activity. These results echoed the findings reported from the test results. The improvement in the test scores test after the teaching intervention provided evidence for the change of the students concerning their interest, attitude, and strategy use in studying English listening skills. Thus, the results obtained from the quantitative data were supported by the interview data.

## Discussion

### Discussion of the Effect of the Blended Learning Mode on Students’ Learning Outcomes

The English listening performance of junior high school students in both the EC and the CC achieved an improvement. However, there was a significant difference in listening achievements between the EC and the CC on the post-test, and a significant variance in the scores before and after the blended teaching intervention in the EC, which was statistically interpreted by the independent samples *t*-test (see results in Table 3; *t*(93) = 7.069^∗^, *p* < 0.05, MD = 3.96). The academic performance of the students in the EC was much better than that of the students in the CC. This finding partially echoes the results of some prior studies, which found that the blended learning mode had a significantly positive impact on the learning outcomes of students (Potter, 2015; González-Gómez et al., 2016). Elsewhere, findings that were inconsistent with the present results showed that a significant decrease in the exam grades of students was observed in the blended courses (Adams et al., 2015; Powers et al., 2016). This difference may have been because of interactions between students, connections between online and offline activities, and the role of the teacher in the blended course. In the present study, a better learning outcome under the blended mode was partially attributed to the appropriate course design in every section. Course design influences the satisfaction of students (Lee, 2014) and their perceived learning (Gray and Diloreto, 2016), which could contribute to good results. When it came to the optimal blended course design, it is worth noting that the teacher was supposed to reasonably arrange both online and offline learning activities before, during, and after the class, with the aim of avoiding posing an excessive burden on students. In addition, it was suggested that the teacher make full use of online learning platforms to collect the feedback of students and identify learning difficulties in understanding micro-lectures and doing listening quizzes, which was conducive to helping the teacher address the problems of students, improve the effectiveness of teaching, and reach the teaching goal in the offline class.

The results of the paired samples tests in Tables 4–7 show that the post-test scores were significantly greater than the pre-test scores on Parts A, B, C, and D of listening comprehension in the EC (*p* < 0.05). The study results revealed that the mean scores of the sub-dimensions of Parts A, B, C, and D in the post-test were all higher than the those of the pre-test. The mean score of Part D among the four parts was the highest, which may be attributed to the higher levels of listening of students to the dialogue and completing the sentences of this dimension. Part C showed the lowest scores, possibly because of the lower level of skills of students in listening to the passage and determining whether the follow-up statements were true or false. This result may have been due to a lack of adequate training of the inferential capability and judgement ability of students within the limited time of the activity or the complexity and the degree of difficulty of inferential tasks. The inferential capability and judgement ability of students were rated low because they used the Quizlet online learning tool to finish tasks without the guidance of the teacher in the first stage. The short time for doing the tasks and intense task design in Quizlet made the students pay little attention to complicated listening skills. Thus, the dimension of Part C did not result in high scores. In contrast, the mean scores of Parts A and B were relatively high, which may be ascribed to the higher levels of skill of students in listening and choosing the right picture, listening to the dialogues, and choosing the best answers to the questions, which were easier and less complicated tasks and aroused interest in students.

The students engaged in doing blended tasks achieved higher scores in listening comprehension, including image-to-text conversion capability, and rapid response capability in a miniature linguistic context, which was examined in Part A; capturing key information, analysing, speculating, and judgement ability in Part B; and capacity for comprehending listening materials and obtaining useful information correctly in Part D. Therefore, they developed higher ability in listening outcomes. These abilities were vital variables in listening comprehension. They correlated highly with the whole listening performance. While doing blended activities, rapid response and capturing key information capability of students could facilitate their analysing and speculating capacity. Moreover, it laid a good foundation for their inferential capability and judgement ability. The listening skills and levels of students in the EC improved a lot after the intervention. Thus, it can be inferred that the effect of the intervention was remarkable. More complex and difficult tasks required more analysing, speculating, inferential capability, and judgement ability to deal with problems at the later stage of doing blended activities. This implies a close relationship between the four kinds of skills in listening comprehension.

The results of the paired samples test in Tables 8–11 depict that the post-test scores were a bit higher compared with the pre-test scores on Parts A, B, C, and D of listening comprehension in the CC. However, when compared with those in the EC, the variances between the pre-test and post-test were not so significant. This result provided evidence for the more positive impact of blended tasks on listening ability of students compared with traditional tasks.

The positive significant correlation (*p* < 0.05) between each part of the listening comprehension in the pre-test and post-test in the EC shown in Table 12 suggests that the students had achieved higher levels and better learning outcomes when engaging with blended activities. However, as shown in Table 13, there was a correlation (*p* = 0.012 < 0.05) of the four parts of A, B, C, and D between the pre-test and post-test in the CC. However, the correlation value of 0.341 indicated a fairly weak relationship in Part C when compared with the other parts. This result implies that the students achieved less positive performance when doing the traditional learning tasks.

As discussed above, students had higher levels of the four skills in listening performance after the intervention. In addition, the results proved that the blended tasks had a more positive impact on the English listening skills and achievement of young learners compared with the traditional ones. Finally, the results of the interview data analysis lend more support to the explanation of the quantitative findings.

### Discussion of the Experiences of Students of the Change in Learning Interest, Attitude and Strategy Use in Doing English Blended Listening Tasks

The learning interest, learning attitude, and strategy use of students in doing English listening activities had undergone changes to become relatively better with the implementation of the blended mode. Different groups of students showed different levels of interest, attitude, and strategy use in different contexts.

Learning interest is the most efficient internal dynamic, and it directly affects learning outcomes of students. The blended tasks emphasised the autonomous learning of learners, and the effectiveness of the autonomous learning of learners mostly depended on their learning interest. No significant differences among the 10 interviewees were found in their interest in doing blended listening tasks. All the interviewees felt inclined to like the multimodal listening tasks, including the videos, micro-lectures, and online discussion community. As Interviewee 3 mentioned, it was more vivid and interesting than merely looking at the pictures in the textbooks and playing the recordings. Other students enjoyed the learning flexibility of the blended mode and various online listening resources. This may be due to a shift in their degree of involvement and interest while they were doing the blended tasks. At any rate, a pleasant and meaningful learning experience, controlled difficulty of the words and contents, appropriate time constraints, and design of listening tasks provided by online tools definitely aroused the interest of students. At the beginning of the class, the teacher explained in detail the goal of learning and introduced steps of tasks so that the students followed her requirements. Thus, the students had a clear goal and confidence in their ability to finish both online and offline listening tasks, which may explain their higher interest in doing online games. The selection of the listening materials, degree of difficulty, and new words were strictly controlled within the scope of the syllabus, so the students experienced a higher level of interest in completing the online games than before the intervention.

According to Ni et al. (2004), “Language learning attitude refers that the learners make their understanding and evaluation of the social value of a language under the influence of social identity, emotions, and other factors.” In line with the conclusions drawn in previous studies (Larson and Sung, 2009; Ma and Lee, 2021; Wang, 2021), and according to the experiences of Interviewees 1–10, this research gave evidence that students clearly took a more positive attitude toward blended learning than they did prior to the intervention. The interviewees all showed pleasure in online activities and felt satisfaction with the achievements, asserting that blended tasks increased their desire to learn English listening skills, which meant they were willing to focus on the task and were able to make use of a variety of strategies. After the intervention, variations in the increase in interest, diversity in strategy use, and focus on different task levels of students may have led to differences in their experience of attitude prior to and after the blended intervention. This may be due to the unique features of the online activity, such as the easy accessibility and availability of course materials, use of web-based apps or electronic tools for real-time communication and thoughtful expressions in the common community, which determined the satisfaction and preference of young learners for the blended mode. Thus, the students showed a change of attitude toward doing the tasks and acquiring listening skills, as well as higher satisfaction. This dimension was positively correlated with interest and strategy use.

Oxford and Nyikos (1989) demonstrated that language learning strategies are specific actions or techniques that students deliberately employ to control, regulate, and enhance their language learning. In the present research, there were differences between the students regarding their strategy use. Although a few interviewees had difficulty in applying more effective strategies in the listening process, most of them had found suitable and efficient learning strategies under the blended mode, which possibly helped improve their listening performance. High achievers in learning performance may be more inclined to manage their learning behaviours strategically to deal with the difficulties they encountered. Several prior studies have shown that language learning strategies are related to the EFL proficiency levels of students, and they have positive effects on each other (Zhang and Xiao, 2006; Rao, 2016). With the pervasive influence of digital technology, the mutual impact was more significant (Oxford and Lin, 2011). Some other research (Richardson et al., 2012) has also suggested that self-regulated learning strategies, such as effort regulation, time management, metacognition, and concentration are relevant to student grade performance in blended mode. However, our study focused on the effect of blended activities on the learning strategy use in English listening comprehension of students, which placed more importance on the dynamic changes of strategies. Under the blended learning mode, students were allowed to choose the materials they were interested in and to use suitable strategies to complete tasks, which may have helped develop their personalised learning methods and autonomous learning ability. Interviewees 9 and 10 were better able to reduce their learning pressure, adopt more effective strategies to correctly identify difficulties, and find solutions to problems than before. Their autonomy helped them to communicate with classmates in the Quizlet discussion community and gain a rich and enjoyable experience. Autonomous learners could manage their learning behaviours and adjust their methods to respond to the requirements of teachers and use diversifying strategies to accomplish tasks. Therefore, they were keen to make more efforts to obtain more skills and optimise their performance. However, differences were found in the experiences of three students compared with the other seven. They held that they did not utilise any new methods, which may have been caused by their lower English level and less autonomy in doing listening tasks. Thus, their sense of experiencing a change in their learning strategy was weaker than that in the other seven students. This difference in the autonomy of strategy use resulted in the differences between students in adopting strategies to do online activities, resulting in an imbalance when it came to enabling a personalised English learning process and employing new methods for the tasks.

## Conclusion and Implications

This study attempted to examine the effect of online and offline integrated teaching on EFL listening performance of young Chinese learners by manipulating an experimental intervention in English language classes using a listening-promotion technical system and tools. The findings of the study revealed significant positive shifts in post-treatment listening competence variables of learners in the treatment group and a moderate positive enhancement in the control group. In addition, the study revealed that the listening performance of students was causally mediated by their perceived motivation, increased interest, triggered positive elevation in attitude, and diversified learning strategies. The participants displayed different extents of experience from the aspects of learning interest, attitude, and strategy use. Specifically, after the intervention, students in the EC obtained significantly higher scores in the four parts of the test (A, B, C, and D) compared with students in the CC. Salient differences were evident between the performance of students in the four listening skills in the EC before and after the intervention. Students showed differences in their perception of experience in strategy use but no variances in the other two dimensions underlying interest and attitude toward blended tasks.

Regarding its theoretical contribution, our study broadens the vision of the online and offline blended mode by not only focussing on the advancement of students in test scores of listening skill and competence but also on such relevant factors as interest, attitude and strategy use throughout their experiences. Previous studies have found that integrating online learning with face-to-face instruction allows teachers to facilitate high-quality teacher–student interaction and enhance the academic outcomes of students (Hastie et al., 2010; Simonson et al., 2012). However, our study further explored change in students in terms of learning interest, attitude, and strategy use, considering the shift to blended learning as a complex experience combining affective and self-regulated process, especially focussing on a specific discipline of EFL listening in the Chinese context. A detailed, multidimensional investigation was conducted on the differences in the sub-dimensions within and between the EC and the CC groups and the different perceptions and complex experiences of the participants, which were scantily inspected in previous studies.

We found that students in the EC experienced increased interest and highly motivated learning, employed new strategies, enjoyed the process, experienced reduced interaction pressure, and achieved success in listening skills while engaging in the blended tasks. These factors were closely related to the listening performance of the students. The experience of the activity of the students was based on their stimulated interest, activated initiative, more positive attitude, and diversified strategies. Therefore, students can improve their English listening competence by completing blended tasks.

With respect to its methodological contribution, adopting a mixed-methods approach, our study empirically validated the practicality of blended-mode-supportive methods in promoting EFL listening of young learners in an actual classroom setting.

Concerning its pedagogical implications, first, this study suggested that teachers should select the personalised online listening tasks and practices most suitable for the language proficiency level of their students, exercising greater flexibility and selectivity to design activities at different stages, namely, before class, during both online and offline class, and after class. Moreover, they should specifically design online game-featured activities, which can efficiently overcome time and space obstacles and maximise the advantages of both modes of teaching to enhance the listening comprehension of students. There was a logical transition from online activity to offline activity and vice versa. Thus, there was a close connection between the three different stages of teaching and learning. Our study found that above all, personalised scaffolding should be provided by the online system or app to give students timely feedback to decrease distraction during task completion (Liu and Song, 2021). Second, teachers should guide students to complete tasks not only through individual inquiry but also through peer-assisted activities and to design more group activities with the aim of stimulating the online cooperation of students, which may provide inspiration for improvement. Moreover, the selection of listening materials should be diversified and involve different topics and genres, but it should also be tailored to meet the needs of students and be within the scope of the linguistic ability of students. Only an appropriate amount and content of listening input could effectively cultivate the comprehensive listening skills of students. Third, more attention should be given to the experiences of students because positive emotions help to stimulate participation and motivation. Teachers should care more about the emotional regulation of learners in online collaborative learning while enacting their coping strategies during the conversion to blended teaching under COVID-19. Teachers should emphasise the task difficulty and the different listening capacities of students, trigger and enhance interest, sustain a positive attitude, and encourage appropriate strategy use. Thus, the design of online activities is supposed to satisfy the needs, enjoyment, and wellbeing of students, decreasing stress and negative emotion. Finally, the role of the teacher needs to change from a controlling or demanding role to that of a facilitator who has trust in students’ ability and guides them in achieving learning goals. Students should be empowered, have more self-directed learning and more involvement in the listening process.

Despite the significant findings, this study has some limitations. As described by some scholars (Osguthorpe and Graham, 2003; Garrison and Kanuka, 2004), it is of great importance to find the “harmonious balance” of “thoughtful integration” between face-to-face and online components. Hence, the teaching design of the blended process in this research needs more improvement and forethought. In addition, because of the limited time and lack of access, the authors were unable to obtain a larger sample to test the reliability and validity of the data. A third limitation is that it did not utilise a questionnaire. In-depth and convincing research on the development of listening ability of learners needs more quantitative methods, such as questionnaires, to be reasonably combined with the qualitative interviews so that the results and effect would be more valid and objective. In addition, in subsequent research, the sampling of participants should be enlarged to involve learners in different types of schools, ages, proficiency levels and educational, social, and cultural backgrounds. Questionnaires could be used and validated, with the items being suitable for the research aim. Moreover, future research efforts should further explore the correlation between online learning and students’ actual English achievements in the Chinese EFL context. With the deep integration of online learning systems and offline curricula becoming a trend in the global educational context, multiple and longitudinal studies and more systematic investigations are needed in the future to shed more light on the study of the online and offline blended mode.

## Data Availability Statement

The original contributions presented in the study are included in the article/Supplementary Material, further inquiries can be directed to the corresponding author.

## Author Contributions

YJ has made substantial contributions to the conception and design of the work. She has designed the theoretical framework, research framework, and research methods of this manuscript. She has drafted and written the first draft of the manuscript and revised it several times critically for important intellectual content, and also revised the final version of this manuscript. YC and JL have helped to search the literature and contributed to writing literature review. YC has organised the database, collected the data, and helped write the first draft of the manuscript. JL has written a few sections of the manuscript and revised the first draft of the manuscript. YW has performed the statistical analysis, has made a great contribution to the revision of the manuscript, to the acquisition, analysis, and interpretation of data for the work. All authors contributed to the article and approved the submitted version.

## Conflict of Interest

The authors declare that the research was conducted in the absence of any commercial or financial relationships that could be construed as a potential conflict of interest.

## Publisher’s Note

All claims expressed in this article are solely those of the authors and do not necessarily represent those of their affiliated organizations, or those of the publisher, the editors and the reviewers. Any product that may be evaluated in this article, or claim that may be made by its manufacturer, is not guaranteed or endorsed by the publisher.

## Supplementary Material

Supplementary Appendix 1 The scoring criteria.

Supplementary Appendix 2 Teaching procedures of the experimental class.

Supplementary Appendix 3 Teaching procedures of the controlled class.
