# Supplementary material for: The Effect of the Online and Offline Blended Teaching Mode on English as a Foreign Language Learners’ Listening Performance in a Chinese Context
Source: Front Psychol. 2021 Nov 16;12:742742. doi: 10.3389/fpsyg.2021.742742 (PMC8634958; doi:10.3389/fpsyg.2021.742742)
Supplement: Supplementary Appendix 1 — The scoring criteria. [file Data_Sheet_1.docx]

**Appendix 1. The Scoring Criteria**

**Shanghai Municipal Senior High School Entrance Examination in 2018**

**English test paper**

**Notes:**

1. The test has 7 parts, a total of 94 questions.

2. The full score of the test paper is 150 points. The test time is 100 minutes.

3. All test questions are numbered consecutively. Please write all your answers in the designated places on the answer sheet, and no points will be given if you do it on the test paper.

**Part I Listening**

**1. Listening comprehension (30 points)**

**A. Listen and choose the right picture (6 points)**


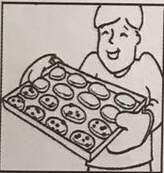

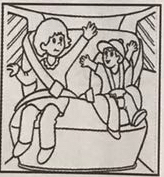

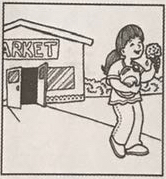

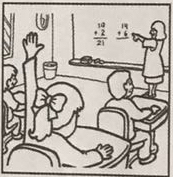


A B C D


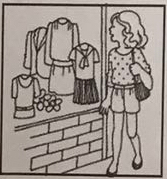

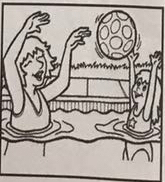

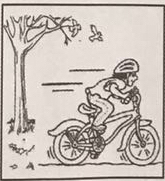

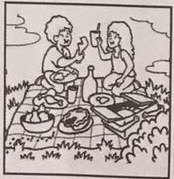


E F G H

1.________2._________3.__________4.__________5.__________6._________

**B. Listen to the dialogue and choose the best answer to the question you hear**

**(8 points)**

7. A) Apple. B) Banana. C) Orange. D) Pear.

8. A) At8:45. B) At 9:00. C) At9:15. D) At 9:00.

9. A) By bus. B) By bike. C) By car. D) By underground.

10. A) Rainy. B) Cloudy. C) Sunny. D) Windy.

11. A) Visit his uncle. B) Visit his classmates.

C) Go to London. D) Go to a language camp.

12. A) In a store. B) At home.

C) At the cinema. D) In a restaurant.

13. A) Husband and wife. B) Doctor and patient.

C) Shop assistant and customer. D) Teacher and student.

14. A) Talk to Lucy. B) Go to the supermarket.

C) Eat more. D) Lose some weight.

**C. Listen to the dialogue and tell whether the following statements are true or false (6 points)**

15. Mary has just become a college student.

16. Simon doesn’t enjoy classroom discussions.

17. Mary has nothing interesting to do after class.

18. Simon will volunteer for an international exhibition in Shanghai.

19. Mary and Simon will go to Shanghai together in five months.

20. Simon gives Mary some suggestions about how to choose a job in China.


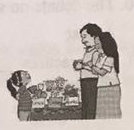
**D. Listen to the passage and complete the following sentences (10 points)**

21. Kitty wanted to plant a _____ ______with beautiful flowers for Mum's birthday.

22. Dad found that the flowers were _______ ______. They were plastic.

23. Dad went to Mr. White _______ _______some flower seeds.

24. Kitty and Dad worked for _______ ______ before Mum came back.

25. After Mum heard the ________ ________ she was moved to tears.

**Shanghai Municipal Senior High School Entrance Examination**

**in 2018**

**Suggested Answers for English Listening Test**

1. **Listening comprehension (30 points)**

1-6 DBAEGF 7-14 CBDAABCD 15-20 TFTTFF

21. little garden 22. not real 23. to borrow 24. 3 hours 25. whole story

**Shanghai Municipal Senior High School Entrance Examination in 2019**

**English test paper**

**Notes:**

1. The test has 7 parts, a total of 94 questions.

2. The full score of the test paper is 150 points. The test time is 100 minutes.

3. All test questions are numbered consecutively. Please write all your answers in the designated places on the answer sheet, and no points will be given if you do it on the test paper.

**Part I Listening**

**I. Listening comprehension（30 points）**

**A. Listen and choose the right picture（6 points）**

**
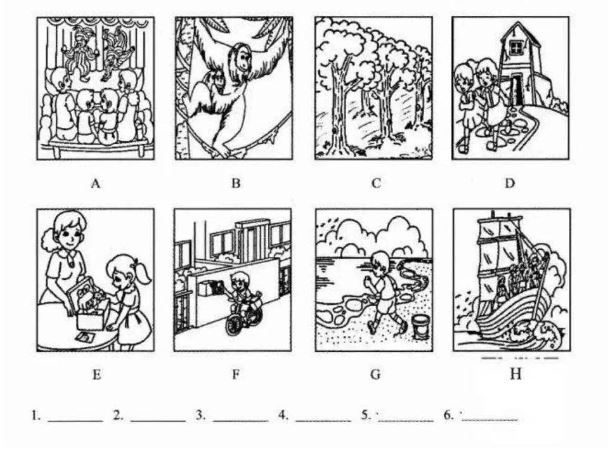
**

1. **Listen to the dialogue and choose the best answer to the question you hear**

**（8 points）**

| 7. | A. A sportsman. | B. A student. | C. A teacher. | D. A doctor. |
| --- | --- | --- | --- | --- |
| 8. | A. History. | B. Physics. | C. Math. | D. Chemistry. |
| 9. | A. Ten years ago. | B. Ten months ago. | C. Two years ago. | D. Two months ago. |
| 10. | A. To give some lectures. | | B. To meet new friends. | |
|  | C. To complete his work. | | D. To improve his memory. | |
| 11. | A. By getting books. | | B. By moving the shelf. | |
|  | C. By picking flowers. | | D. By watching the door. | |
| 12. | A. At the restaurant. | | B. In the kitchen. | |
|  | C. At the supermarket. | | D. In the garden. | |
| 13. | A. The Internet. | B. The subjects. | C. The price. | D. The levels. |
| 14. | A. He’s afraid his son will fall asleep in writing lessons. | | | |
|  | B. He thinks the programmes will take too much time. | | | |
|  | C. He’s glad that reading before sleep in his son’s habit. | | | |
|  | D. He believes after-school lessons are necessary to attend. | | | |

**C. Listen to the passage and tell whether the following statements are true or false（6 points）**

15. There was a fence between Charlie’s garden and the neighbor’s.

16. The football hit the cat heavily and it made a loud sound.

17. The whole flower pot was broken into pieces by the football.

18. Charlie climbed over the wall to the neighbor’s garden to get his football.

19. Charlie put the broken pieces into his pocket before he left the garden.

20. From the story, we know that Charlie finally chose to be honest.

**D. Listen to the dialogue and complete the following sentences（10 points）**

21. Bob thinks that dropping a little piece of plastic is not a __________ __________.

22. Rubbish will hurt the environment and __________ __________ from the park’s natural beauty.

23. Bob has seen lots of litter in some of the neighborhoods __________ __________.

24. The public place would __________ __________ if everyone dropped rubbish them.

25. On Bob and Jessie’s way home, they’ll walk past a rubbish bin near the __________ __________.

**Shanghai Municipal Senior High School Entrance Examination**

**in 2019**

**Suggested Answers for English Listening Test**

**I. Listening comprehension (30 points)**

1-6 BEAHGC 7-14 DACDABCB 15-20 TFFFTT

21. big deal 22. take away 23. around town 24. be dirty 25. bus stop

**Appendix 2. Teaching Procedures of the Experimental Class**

**The design of online learning procedures (before class)**

---The Adventure of Tom Sawyer

**Step 1:** Log in the Quizlet website and watch the micro-lecture video clip of this topic.

**Step 2:** Complete a listening quiz, which includes matching the words with their

corresponding pronunciations, repeating the words in the micro-lecture, blank-filling, and multiple choice quetions.

**Step 3:** You are asked to write down your difficulties in understanding the main content and words in the micro-lecture and in doing the listening quiz. Based on your feedback, your learning problems will be dealt with in the offline teaching.

**Step 4:** Design your study plans, raise your questions and express your opinions in the online discussion community.

**Step 5:** Check your learning feedback on the website.

**The design of offline teaching procedures (during class)**

---The Adventure of Tom Sawyer

**Step 1: Lead-in**

The teacher plays the video clip about Mark Twain and then asks several students to introduce him further.

**Step 2: Review and Introduction**

The teacher has a review of the results of students’ online study, and then helps deal with students’ learning difficulties and solve the remained problems based on the online assessment. Then the teacher will explain the teaching goal and teaching focus of this lesson..

**Step 3: Script Analysis and Group work**

The teacher plays the recording of the text, analyses the script, the characters and plot of the story, and asks students to listen to it for two times and to complete the tasks designed based on students’ listening learning scores on the Quizlet website. The tasks include finishing multiple choice questions, blank-filling, and answering questions, requiring students to cooperate with group members to complete listening tasks.

**Step 4: Extension**

The teacher asks students to listen to another clip of recording which is related to the main topic and to answer the questions independently.

**Step 5: Practice and Presentation--- Role play**

The teacher will create situations for students to use the language learned just now. Students are divided into different groups and play the roles in the story.

**Step 6:** **Reflection and Consolidation---Share time**

Section A: Expressing congratulations and sympathy

Section B: Students are asked to share their favorite stories about Tom Sawyer which are presented in the listening materials on the Quizlet platform.

**Step 7: Summary and Homework.**

The teacher leads students to summarise new words, expressions and key information in the listening materials, and assigns the homework.

**The design of online revision tasks (after class)**

**Step 1:** Log in the Courseware APP and complete the listening homework.

**Step 2:** Write down your answers to a relevant short discourse displayed in the online system within 3 minutes, then upload them to the Quizlet system.

**Step 3:** Listen to the daily news on the courseware APP.

**Appendix 3. Teaching Procedures of the Controlled Class**

---The Adventure of Tom Sawyer

**Pre-listening**: Warming up

The teacher shows the pictures of Mark Twain and then asks one or two students to introduce Mark Twain to classmates, and the whole class predict the characters and the plot of the story based on the title and pictures on Page 106.

**While-listening**: Presentation

The teacher plays the recording of of the text, analyses the characters and plot of the story, and asks students to listen to it for two times and to complete the tasks including matching, blank-filling, answering questions, and finishing multiple choice questions.

Then the teacher asks the whole class to listen to a clip of recording about a jumping frog and to choose the correct answers to the questions. Then the teacher asks the students to listen to it again and complete the task of putting the pictures in the correct order, finishing multiple choice questions, and blank-filling..

**Post-listening**: Practice

The teacher asks students to retell the story in 5 minutes, and requires the members of groups to play the roles in the story, and then retell their favorite stories about Tom Sawyer.
